# Whole-Genome Sequencing of Two *Bartonella bacilliformis* Strains

**DOI:** 10.1128/genomeA.00659-16

**Published:** 2016-07-07

**Authors:** Yolanda Guillen, Maria Casadellà, Ruth García-de-la-Guarda, Abraham Espinoza-Culupú, Roger Paredes, Joaquim Ruiz, Marc Noguera-Julian

**Affiliations:** a irsiCaixa AIDS Research Institute, Badalona, Catalonia, Spain; b Universitat Autònoma de Barcelona, Bellaterra, Catalonia, Spain; c Laboratorio de Microbiología Molecular y Biotecnología, Facultad de Ciencias Biológicas, Universidad Nacional Mayor de San Marcos, Lima, Peru; d Universitat de Vic–Universitat Central de Catalunya, Vic, Catalonia, Spain; e Unitat VIH, Hospital Universitari Germans Trias i Pujol, Badalona, Catalonia, Spain; f ISGlobal, Barcelona Centre for International Health Research (CRESIB), Hospital Clínic, Universitat de Barcelona, Barcelona, Spain

## Abstract

*Bartonella bacilliformis* is the causative agent of Carrion’s disease, a highly endemic human bartonellosis in Peru. We performed a whole-genome assembly of two *B. bacilliformis* strains isolated from the blood of infected patients in the acute phase of Carrion’s disease from the Cusco and Piura regions in Peru.

## GENOME ANNOUNCEMENT

Carrion’s disease is a vector-borne neglected illness restricted to Andean valleys in Peru, Ecuador, and Colombia (1), where it remains endemic. The causal agent of Carrion’s disease is *Bartonella bacilliformis*, an intracellular, nonfermentative, pleomorphic, Gram-negative microorganism, with *Lutzomyia verrucarum* being the main illness vector.

This illness has two different phases. In the acute phase, called Oroya fever, the intense destruction of erythrocytes leads to a severe hemolytic anemia and temporal immunosuppression, which facilitates both the reactivation of silent illnesses, such as tuberculosis or histoplasmosis, and the growth of opportunistic infections that may be fatal, such as bloodstream *Salmonella* infections (1, 2). In fact, in the preantibiotic era, Oroya fever was considered one of the most lethal infections, having a mortality rate of up to 85% (3); at present, a 10% mortality rate is reported in reference hospitals, even with correct antibiotic treatments (1).

The chronic phase is considered to take place months after Oroya fever, due to the development of partial immunity, but it may also be present even without a previous acute diagnosis. This phase is characterized by the proliferation of verrucous lesions, so-called verruga peruana. In this phase, the most serious complication is the presence of verrucous lesion bleeding, which in extreme cases may require blood transfusions (4).

Often, a clinical cure does not result in microbiological clearance, leading to asymptomatic carriers, with persistent *B. bacilliformis* bacteremia serving as a source for human-to-human transmissions (1). Nonetheless, their real number remains uncertain due to the lack of enough sensitive diagnosis tools (5); additional genomic data may help close these gaps.

Sequencing of the two *B. bacilliformis* strains, USM-LMMB-006 and USM-LMMB-007, collected in 2011 in southern (La Convención, Cusco) and northern Peru (Huancabamba, Piura), respectively, and identified as causal agents of Oroya fever (6), was carried out at the genomics platform of the Germans Trias i Pujol Research Institute using an Illumina MiSeq sequencer with a paired-end 300-bp sequencing kit. Using Trimmomatic (7), low-quality reads were filtered out and adapter sequences were trimmed. Quality-controlled sequence reads were assembled using A5-miseq (8) small genome assembly software (8). Protein-coding genes were predicted using the NCBI Prokaryote Genome Annotation Pipeline (PGAP) (9). The functions of the predicted protein-coding genes were annotated with the Clusters of Orthologous Groups (COG) (10) database using the WebMGA (11) interface with standard parameters. For strains USM-LMMB-006 and USM-LMMB-007, assemblies produced 15 and 11 scaffolds with median depths of coverage of 72× and 77×, total sizes of 1,401,011 and 1,405,613 bp, and GC contents of 38.0% and 38.0%, respectively. The draft genomes of *B. bacilliformis* strains USM-LMMB-006 and USM-LMMB-007 contained 1,136/1,135 coding sequences, 3/3 rRNAs, and 39/39tRNAs, respectively.

Using COG functional assignment, the 996 (88%) and 998 (88%) predicted proteins of strains USM-LMMB-006 and USM-LMMB-007, respectively, could be classified into 868 COG families and 21 COG classes. The most abundant COG classes in both strains were related to translation, ribosomal structure, and biogenesis, including 135 predicted proteins in both strains. The abundant classes in USM-LMMB-006 and USM-LMMB-007 were general function (92/92); replication, recombination, and repair (76/74); amino acid transport and metabolism (76/76); and energy production and conversion (72/72); 88/86 had unknown function.

### Nucleotide sequence accession numbers.

This whole-genome shotgun project has been deposited at NCBI GenBank under the sequence accession numbers LQWW00000000 (LQWW01000001 to LQWW01000015) and LQXX00000000 (LQXX01000001 to LQXX01000011) for strains USM-LMMB-006 and USM-LMMB-007, respectively.
